# Metrics of perineal support (MOPS) study

**DOI:** 10.1186/s12884-020-03042-3

**Published:** 2020-06-11

**Authors:** Vladimir Kalis, Zdenek Rusavy, Linda Havelkova, Tomas Zitka, David Tolar, Khaled M. Ismail

**Affiliations:** 1 Department of Gynecology and Obstetrics, University Hospital, alej Svobody 76, 304 60 Pilsen, Czech Republic; 2 Biomedical Center, Faculty of Medicine in Pilsen, Charles University, Pilsen, Czech Republic; 3 New Technologies - Research Centre, University of West Bohemia, Pilsen, Czech Republic

**Keywords:** Keys: Manual perineal protection, Physiology, Vaginal delivery, Computational modelling, Finnish method, Pressure, Forces, Duration, Variability

## Abstract

**Background:**

Manual perineal protection (MPP) is an intrapartum intervention suggested to protect perineal integrity during childbirth. Proper execution of MPP is complex and evaluation of its true contribution is difficult in the clinical setting because of the large number of obstetric variables, some of which are hardly quantifiable. In this study we aimed to gather initial data on the forces executed by the accoucheur’s thumb, index and middle fingers during MPP at the time of fetal head expulsion, quantify the duration of the intervention and investigate the timely interaction of the different components of MPP.

**Methods:**

Two bespoke right-handed measurement gloves (MG), with built in sensors, were designed and produced. The MG allowed the electronic real-time measurement of applied forces during MPP and transferred this data wirelessly to an integrated computer system. Sterile gloves were worn over the MG when used at the time of birth. The study was undertaken between January and December 2019. Singleton, term pregnant women having their first vaginal birth who provided a valid written consent were enrolled into this prospective pilot study. All deliveries were undertaken by one of two obstetricians experienced in MPP.

**Results:**

Twenty women were enrolled. The mean duration of execution of MPP during the last contraction was 13.6 s. In 20% it lasted < 5 s. The overall mean values of the average and maximum forces of the thumb, index and middle fingers were 26.7 N; 25.5 N; 20.2 N and 34.3 N; 32.6 N; and 27.6 N respectively. The onset of fingers and thumb activity was simultaneous in 13 cases (65%), while in seven (35%) deliveries the middle finger’s force activity was initiated later.

**Conclusions:**

MPP during fetal head expulsion happens over a short period of time. In the majority of cases the thumb and fingers actions started simultaneously. There were differences in the duration of application and the forces executed by the fingers and thumb between the two practitioners, however this was only significant for thumb measurements. The results obtained will aid in improving further MPP modeling studies to optimize the technique.

## Background

Birth-related perineal trauma can be a cause of longstanding significant adverse effect on a woman’s quality of life [1–3]. Manual perineal protection (MPP) was historically suggested to protect the integrity of the perineum during fetal head expulsion [3–6]. However, more recent RCTs brought doubts as to its real effectiveness in reducing such trauma [7, 8]. In contrast, larger well-designed prospective studies have consistently shown a significant positive contribution of MPP to preserve perineal integrity and reduce risk of high order tears [9–16].

Effective MPP is a complex procedure to execute [17–21]. Indeed, a recent survey of practitioners from units that support a routine MPP policy demonstrated that only 5.6% of respondents were able to provide a comprehensive description of MPP correctly [18, 19]. The Finnish technique of MPP (FMPP) involves application of the thumb and index finger of the dominant hand on the perineal skin anterolateral to the fourchette and their approximation to reduce midline perineal strain. The flexed middle finger is used to apply pressure against the perineal body to facilitate the process of fetal head extension. While, the non-dominant hand controls the speed of fetal head expulsion and facilitates fetal head extension [18, 19]. Practically, it is difficult to evaluate the actual contribution of the individual components of FMPP in a clinical setting, because of a high number of confounders and obstetric variables, some of which are hardly quantifiable. Moreover, a proper timely execution of this procedure is challenging since the process of fetal head expulsion happens during a relatively short and critical time.

To overcome the difficulties of real-life clinical evaluation, a simplified computational finite elements model has been used as surrogate [17, 20, 21]. This has enabled to optimize the placement and direction of movement of the different components of FMPP that was associated with the lowest degree of perineal strain [17, 20, 21]. Nonetheless, computational modeling studies are limited by the lack of realistic information about the actual duration of active MPP, the dynamic timeline of when the different individual components are utilized in an actual birth and the forces applied at their execution. We believe that understanding such parameters would improve the precision of future computational modeling and provide objective means to refine the technique further. Therefore, in this study we aimed to gather initial data on the forces executed by the accoucheur’s thumb, index and middle fingers at the time of MPP, quantify the precise duration of the intervention, investigate the timely interaction of the different components of MPP and explore the degree of intra and inter-practitioner variability in the above variables.

## Methods

**Fig. 1:**
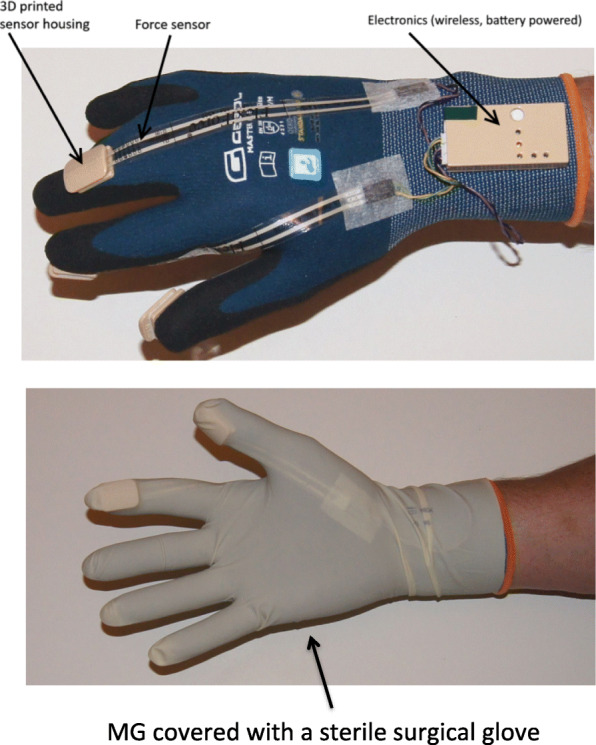
The measurement glove

For the purpose of this study, two bespoke identical right-handed measurement gloves (MG) were designed and produced at New Technologies - Research Centre, University of West Bohemia, Pilsen, Czech Republic Fig. 1.

MG is an electronic measurement device allowing the measurement of forces applied during MPP. It is part of an integrated measurement system, which collects, transfers and archives measurement data on a wirelessly connected PC. The glove has three force sensing elements located in pre-selected positions relating to points on the accoucher’s dominant hand, which are in contact with maternal perineum and exert the main pressure force at the time of MPP. The MG and its data acquisition system were designed to the highest standards for safety and durability. The glove is powered by a miniature 3 V lithium cell battery and uses a wireless low power data transmission with the electronics electrically isolated from the glove user and the touched surface. Before each use the glove was covered by disposable sterile surgical glove. The MG’s built-in force sensors (Tekscan, Inc., 307 West First Street South Boston, MA 02127, US) have a maximum allowed force application of 100 N/sensor with a frequency acquisition of 100 samples per second. The system was made very simple to operate with.

Measured data were transferred wirelessly at the 2.4 GHz ISM (industrial, scientific and medical) frequency band. The used communication protocol is compliant with IEEE 802.15.4 standard providing high reliability of data transmission. Used wireless communication method was verified in a previous medical sensor project and was proven safe for use in medical sensors located in close proximity to the human body [22].

Following several test measurements on a childbirth simulation model, the clinical measurements were performed on the labor ward of a tertiary perinatology care unit, department of Gynecology and Obstetrics, University Hospital, Pilsen, Czech Republic. Woman at term having their first spontaneous vaginal birth with good understanding of Czech language and who signed a valid consent were considered eligible for the study. The local Ethics committee approved this study on October 4, 2018 and all women signed an informed consent.

At the time of birth, the accoucher wore the MG, which was completely covered by a surgical sterile glove. Signal from the sensors was filtered by low-pass Buttherworth filter with a cut-off frequency of 1 Hz to remove noise. The course of the whole end of the second stage of labor was recorded. Simultaneous voice recording was done to avoid error in data interpretation. Data from the last contraction, the fetal head expulsion, were evaluated and compared.

Inter-individual variations in applied forces were evaluated with non-parametric ANOVA (2-sample Kruskal-Wallis test) using Python 3.7 and SciPy 1.1.0 statistical package. The cut-off for statistical significance was set at *p* < 0.05. For intra-individual differences, we relied on standard deviations and interquartile ranges to describe the variation.

## Results

Between January 1 and December 31, 2019 two experienced obstetricians, actively involved in delivering MPP structured training, attended 20 term first vaginal deliveries (10 VK and 10 ZR). Full data were available from all measurements. There were no operative vaginal deliveries or episiotomies performed in this cohort. None of the study participants sustained obstetric anal sphincter injuries at the time of birth. The demographic data and obstetric variables of the cohort are presented in Table 1.

**Table 1:** Obstetric data of the study group

Procedure - independent characteristics	Median (range 25–75%)	Mean ± SD
Maternal age, y	29.0 (26.0–30.0)	29.2 ± 5.9
Body mass index^a^	29.6 (26.1–32.2)	29.8 ± 4.3
Duration of the 2nd stage of labor, min	35.5 (15.3–49.0)	37.8 ± 27.2
Head circumference, cm	34.5 (33.9–35.1)	34.5 ± 1.2
Perineal trauma, degrees	1 (0–1)	0.9 ± 0.8
Neonatal weight, g	3560 (3418–3875)	3549 ± 378
Apgar score at 1 min	9 (9–10)	9.1 ± 0.9
Apgar score at 5 min	10 (9–10)	9.7 ± 0.6
Neonatal pH	7.29 (7.26–7.36)	7.29 ± 0.09

^a^Calculated as weight in kilograms divided by the square of height in meters

The mean duration of execution of MPP at the time of fetal head expulsion was 13.6 s, the overall mean values of the average forces of the thumb, index and middle fingers were 26.7 N; 25.5 N; and 20.2 N while the means of maximum forces were: 34.3 N; 32.6 N; and 27.6 N respectively (Table 2). In 13 cases (65%) the activity of all digits started simultaneously while in seven (35%) deliveries the middle finger’s activity was initiated later. However, in only three (15%) cases this delay was longer than 1 s.

**Table 2:** MPP measurement data

MPP variable	Accoucheur	Mean ± SD	Median (interquartile range)	*p*^a^
Duration, s	Overall	13.6 ± 8.2	13.6 (8.8–15.4)	
	A 1	12.5 ± 5.7	12.5 (10.3–14.1)	.76
	A 2	14.7 ± 10.4	14.1 (6.9–17.2)	
Thumb mean force, N	Overall	26.7 ± 6.8	26.1 (20.3–31.8)	
	A 1	30.5 ± 6.5	30.0 (26.7–34.8)	.008
	A 2	22.9 ± 4.8	21.9 (19.3–25.6)	
Index finger mean force, N	Overall	25.5 ± 5.7	26.8 (22.0–29.6)	
	A 1	27.7 ± 5,7	28.3 (26.3–30.0)	.06
	A 2	23.3 ± 5.0	22.3 (21.8–26.8)	
Middle finger mean force, N	Overall	20.2 ± 7.8	20.5 (13.7–26.2)	
	A 1	18.1 ± 7.7	20.2 (11.9–24.2)	.23
	A 2	22.2 ± 7.8	23.1 (14.5–27.8)	
Thumb maximum force, N	Overall	34.3 ± 7.2	35.1 (28.6–38.7)	
	A 1	37.8 ± 7.0	38.1 (32.5–42.5)	.03
	A 2	30.9 ± 5.7	29.8 (25.7–35.9)	
Index finger maximum force, N	Overall	32.6 ± 6.6	33.1 (27.3–38.6)	
	A 1	34.6 ± 5.7	33.9 (32.3–38.4)	.17
	A 2	30.4 ± 7.0	27.9 (25.5–37.1)	
Middle finger maximum force, N	Overall	27.6 ± 10.1	27.0 (21.8–33.0)	
	A 1	23.9 ± 8.7	24.7 (18.6–31.5)	.20
	A 2	31.2 ± 10.6	29.7 (24.4–39.0)	

*A1* Accoucher 1, *A2* Accoucher 2

^a^ non-parametric ANOVA (2-sample Kruskal-Wallis test) of medians

**Fig. 2:**
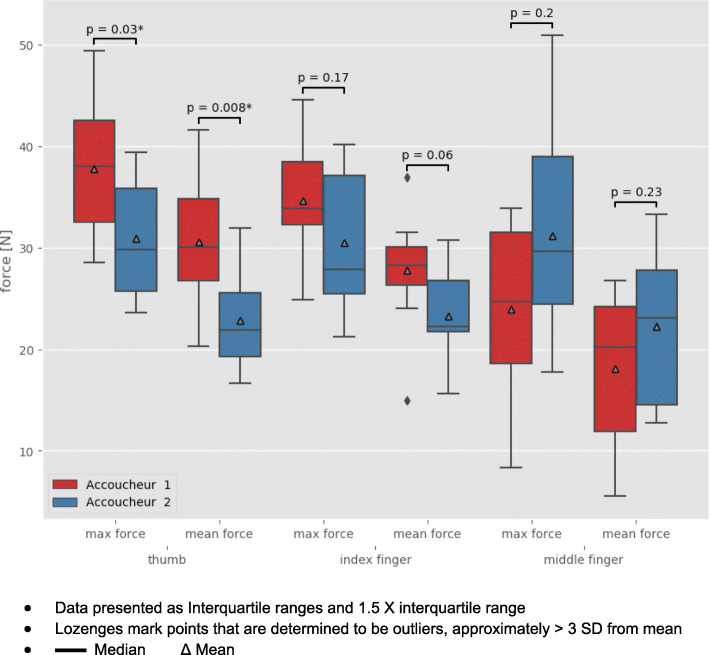
Mean and maximum forces applied by the thumb, index and middle fingers

There were statistically significant differences in the mean (*p* = .008) and maximum (*p* = .03) thumb forces between the two obstetricians. However, the differences in MPP duration and forces executed by the index or middle fingers remained non-significant despite the variation in the forces applied by the individual practitioners in the different births (Table 2, Fig. 2).

Irrespective of the accoucheur, there were strong correlations between mean and maximum forces of the thumb (0.95), index finger (0.91) and middle finger (0.89).

## Discussion

### Main findings

In this study we were able to quantify, in real time, the forces executed by two independent accoucheurs at the time of FMPP. The bespoke system enabled us to capture data for the extent of pressure applied by the thumb, index and middle fingers during the intervention and to assess the inter- and intra-practitioner variability in performing MPP. The duration of the active FMPP intervention was shorter than our expectation where in 6 births (30.0%) it lasted <10s and in 4 (20.0%) births the intervention took < 5 s. This highlights the importance of the timing and precision by which FMPP must be executed to ensure its effectiveness during fetal head expulsion. In the majority of deliveries the thumb, index and middle fingers activity started almost simultaneously with the maximum forces applied at a median of 5.7–5.9 s after FMPP initiation. Interestingly data relating to the duration of the active intervention and the interaction between its components was fairly consistent amongst the 2 practitioners and in the different births. Moreover, apart from thumb measurements, there were no significant differences in the force applied by the index and middle fingers between the participating obstetricians. Although not formally assessed in the study, it is possible that the observed differences in thumb measurements are a reflection of the heterogeneity of maternal, fetal and labor related factors that influence the birth process. Many of these variables, like the strength of the uterine contraction or maternal pushing, are not easily quantifiable to perform an adequate scientific evaluation. It is also possible that this variation reflects the actual differences in how practitioners perform the technique. This is potentially important because it could be linked to variation in reported effectiveness of the intervention.

### Comparison to other studies

Our group has previously demonstrated that, during FMPP, the optimal placement of the thumb and index finger is 2 cm anterior of the fourchette and 12 cm apart. While the most effective distance and direction of movement is to approximate these digits by a distance of 1 cm on either side without changing their antero-posterior orientation to the fourchette [17, 20, 21, 23]. Additionally, it has been also shown, that an imprecision of just 1 cm might increase the maximum strain at the perineum by up to 30% [20]. This highlights the potential magnitude of the effect of, what could be perceived, as a subtle variation in technique performance. To our knowledge this is the first study of the metrics of manual perineal support. We believe that this information will complement the findings of our previous studies in the field. Indeed, the results obtained provide useful information that will feed into our refined computational model with the aim of refining the technique to maximize its effectiveness. This will ultimately provide invaluable information for structured training programs and clinical practice [24].

### Strengths and limitations

We recognize that some aspects of this work can be perceived as limitations. Firstly, we only captured data from two practitioners. However, because our primary intention was to use this information as a benchmark to feed into our computational model, it was essential for us to gather data from senior obstetricians, experienced in performing FMPP and fully aware of its components. In addition, this allowed us to evaluate the intra-practitioner variability. Secondly, we are aware that our sample size is fairly small. Nevertheless, we mainly wanted to collect pilot data on the range of forces applied when performing this technique and the timings related to the active intervention. In contrast, the development of a specifically designed integrated system to capture the required data and the fact that this is the first report of such measurements are major strengths of our study.

## Conclusion

The duration of active application of MPP at the time of head expulsion lasted for only a very short duration. In the majority of cases the thumb and fingers actions start simultaneously. To our knowledge this is the first study quantifying the forces of the accoucheur’s fingers on the perineum as well the duration of MPP itself and the timely involvement of the middle finger of the dominant hand. The results obtained are important to improve the numerical modeling of MPP in order to produce more realistic data.

## Data Availability

The datasets used and/or analysed during the current study are available from the corresponding author on reasonable request.

## Abbreviations

FMPP: Finnish technique of manual perineal protection
MG: Measurement glove
MPP: Manual perineal protection
